# Draft genomes of *Salmonella* Dublin from North Dakota State University veterinary diagnostic laboratory

**DOI:** 10.1128/mra.00636-25

**Published:** 2026-03-04

**Authors:** Sophia M. Kenney, Kelli J. Maddock, Erika Ganda

**Affiliations:** 1 Department of Animal Science, The Pennsylvania State University 8082, University Park, Pennsylvania, USA; 2 Molecular, Cellular, and Integrative Biosciences Graduate Program, Huck Institutes of the Life Sciences, The Pennsylvania State University 8082, University Park, Pennsylvania, USA; 3 One Health Microbiome Center, Huck Institutes of the Life Sciences, The Pennsylvania State University 8082, University Park, Pennsylvania, USA; 4 Veterinary Diagnostic Laboratory, North Dakota State University 3323, Fargo, North Dakota, USA; University of Maryland Baltimore, Baltimore, Maryland, USA

**Keywords:** *Salmonella*, genome, pathogen

## Abstract

We report 26 high-quality draft genomes of *Salmonella enterica* subsp. *enterica* serovar Dublin isolated from clinical case submissions to the North Dakota State University Veterinary Diagnostic Laboratory. Hybrid assemblies were generated *de novo,* ranging from 2 to 12 contigs, with an average of 52.15 GC% and 99.55% completeness.

## ANNOUNCEMENT

*Salmonella enterica* serovar Dublin is a clinically significant bovine pathogen and one of the most frequently isolated serovars from dairies in the United States (1). Transmission dynamics and pathogen evolution may be better understood through genomic analyses. To this end, we identified 26 *S*. Dublin strains isolated from submissions to the North Dakota State University (NDSU) Veterinary Diagnostic Lab for whole-genome sequencing.

Samples were received as part of a routine diagnostic investigation. Culture was performed by directly plating tissue or feces onto sheep’s blood and Tergitol 7 agar (Hardy Diagnostics, USA) and tetrathionate broth (Remel, Thermo Fisher, USA) and aerobic incubation at 35°C in ambient conditions for 24 h. If no *Salmonella* was identified at 24 h, tetrathionate broth was subcultured to Tergitol 7 and XLT-4 (Xylose-Lysine-Tergitol 4; Hardy Diagnostics, USA) agar and incubated as before. Red colonies on Tergitol 7 and pink-yellow or black colonies on XLT-4 were screened and identified by matrix-assisted laser desorption ionization time-of-flight mass spectrometry (Bruker Biotyper, Bruker Daltonics, USA). The National Veterinary Services Laboratory (Ames, IA) performed serotyping and confirmed the isolates as *S*. Dublin (2). Penn State University performed sequencing preparation, analysis, and assembly as follows.

DNA extraction from broth cultures, brain heart infusion (BHI; RPI Corp, USA) broth inoculated from frozen stock and incubated overnight at 37°C, was performed as outlined in Table 1 at https://github.com/sophiakenney/ndsu_dublin_mra/blob/main/Table1.xlsx, according to the manufacturer’s instructions. Library preparation was performed at Novogene Corporation Inc (Sacramento, USA), where DNA was randomly sheared prior to adapter ligation, fragment size selection, PCR, and sequencing (see Table 1 at https://github.com/sophiakenney/ndsu_dublin_mra/blob/main/Table1.xlsx). For long-read sequencing, DNA extraction from BHI broth cultures was performed according to the manufacturer’s instructions using methods outlined in Table 1 at https://github.com/sophiakenney/ndsu_dublin_mra/blob/main/Table1.xlsx. Uniquely indexed SMRTbell templates from each sample were created and then pooled in equimolar concentrations for sequencing (Table 1 at https://github.com/sophiakenney/ndsu_dublin_mra/blob/main/Table1.xlsx). Shearing and size selection were performed.

For Illumina reads, raw sequence quality was evaluated as previously described (3). PacBio raw reads were converted from BAM to FASTQ files using pbtk (v3.5.0) (4), followed by quality evaluation as above. Sequencing depth was determined using filtered short and long reads using *S. enterica* Typhimurium LT2 reference strain (5). Hybrid genomes were assembled *de novo* with Unicycler (v0.5.0) in normal mode (6). Assembly quality was evaluated with QUAST (v5.2.0) (7) and CheckM (v1.2.2) (8) prior to submission and annotation (9). All sample metadata, sequencing preparation and output, and assembly and annotation details are reported in Table 1 at https://github.com/sophiakenney/ndsu_dublin_mra/blob/main/Table1.xlsx.

## Data Availability

All data/code are available in the paper or https://github.com/sophiakenney/ndsu_dublin_mra.
